# Onlay versus Inlay Lower Trapezius Tendon transfer for posterosuperior functional irreparable rotator cuff tears: a 2-year clinical and imaging study

**DOI:** 10.1016/j.clinsp.2026.100934

**Published:** 2026-04-14

**Authors:** Cheng-Pang Yang, Tung-Lung Wu, Louis Yi Lu, Lei Hsia, Poyu Chen, Joe Chih-Hao Chiu

**Affiliations:** Linkou Chang Gung Memorial Hospital, Gueishan District, Taiwan

**Keywords:** Massive, Irreparable, Lower Trapezius Tendon transfer, Onlay, Inlay

## Abstract

**Background:**

This study compared the clinical and radiological outcomes of Onlay and Inlay Lower Trapezius Tendon (LTT) transfer techniques in managing posterosuperior Functional Irreparable Rotator Cuff Tears (FIRCTs).

**Methods:**

This retrospective comparative study included patients who underwent Onlay and Inlay LTT transfer. The inclusion criteria were FIRCTs defined by tendon retraction to the glenoid, fatty infiltration > 2 grade according to Goutallier, and with a positive External Rotation (ER) lag sign. Patients with irreparable subscapularis, dysfunctional deltoid, advanced glenohumeral osteoarthritis, cuff tear arthropathy, and revision surgeries were excluded. Outcomes measures included the active Range Of Motion (ROM), Acromiohumeral Distance (AHD), American Shoulder and Elbow Surgeons (ASES) score, Single Assessment Numeric Evaluation (SANE) score and Constant score.

**Results:**

The study included 39 patients (21 Onlay and 18 Inlay LTT) with at least 2-year follow-up. Both groups achieved significant improvement in active forward elevation from 118.6 ± 57.6° to 145.7 ± 18° in the Onlay group (*p* = 0.046) and 112.2 ± 49.2° to 140.6 ± 30.6° in the Inlay group (*p* = 0.046). The ER improved from 31 ± 21.2°to 44.3 ± 16° in the Onlay (*p* = 0.027) and 29.4 ± 27.1° to 45 ± 16.9° in the Inlay group (*p* = 0.046). The postoperative AHD was better in the Onlay group (7.3 ± 2.5 vs. 4.8 ± 3.2 mm, *p* = 0.001). The ASES, SANE, and Constant scores all improved significantly at final follow-up (all *p* < 0.001) in both groups and reached the Minimal Clinically Important Difference.

**Conclusion:**

Both the onlay and Inlay techniques of LTT transfer effectively improved shoulder function in patients with irreparable posterosuperior rotator cuff tears. The Onlay technique resulted in better AHD at final follow-up.

## Highlights

• Both Onlay and Inlay techniques of LTT transfer effectively improved shoulder function in patients with irreparable posterosuperior rotator cuff tears.
• The Onlay technique resulted in better AHD at the final follow-up.
• This study provides evidence to guide surgical decision-making of the Onlay technique for LTT transfer in this patient population.

## Introduction

For patients with massive, irreparable Rotator Cuff Tears (RCTs), a range of surgical treatments has been proposed to improve shoulder function and to alleviate associated pain, including partial repair, long head of the biceps tenodesis/tenotomy, Superior Capsule Reconstruction (SCR), subacromial balloon spacer, synthetic augmentation, Reverse Shoulder Arthroplasty (RSA), and open-/arthroscopic-assisted tendon transfers.^1^, ^2^, ^3^, ^4^, ^5^, ^6^, ^7^, ^8^, ^9^, ^10^, ^11^, ^12^ Among them, tendon transfers have emerged as a promising reconstructive option to rebalance the anterior-posterior force couple.^13^, ^14^, ^15^

In recent years, Lower Trapezius Tendon (LTT) transfer has gained increasing attention as a treatment option for posterosuperior Functional Irreparable Rotator Cuff Tears (FIRCTs).^16^ This technique, initially used with success in patients with brachial plexus injuries to increase the External Rotation (ER) torque,^17^ has shown excellent clinical and radiographic outcomes in recent series of patients with FIRCTs.^13^, ^18^, ^19^ LTT transfer has demonstrated promising results in restoring shoulder function and reducing pain, making it an increasingly considered alternative for patients who are not ideal candidates for other surgeries, such as RSA. However, the optimal method of performing the LTT transfer is still an ongoing debate.^16^

In this study, the authors compared two common variations of LTT transfer, the “Onlay”^18^ and “Inlay”^20^ techniques. The “Onlay” technique involves placing an Achilles tendon allograft onto the footprint of the cuff and securing it with suture anchors and augmenting it medially with autologous LTT.^18^ The “Inlay” technique involves creating a bone tunnel from the insertion site of infraspinatus posteriorly, to the bicipital groove anteriorly, inside the humeral head, through which the autologous semitendinosus or peroneal longus tendon graft is driven and then secured medially with autologous LTT.^20^

This study was designed to directly compare two common variations of LTT transfer: the “Onlay” and “Inlay” techniques. The primary clinical question was: In patients with FIRCTs, does the Onlay technique lead to superior clinical outcomes compared to the Inlay technique? The authors hypothesized that the Onlay technique, which uses an Achilles tendon allograft placed onto the cuff footprint, would be associated with significantly better Patient-Reported Outcome Measures (PROMs) than the Inlay technique. The authors postulate this is due to the Onlay method's ability to create a greater spacer effect in the subacromial space. This investigation aims to provide critical data to help resolve the ongoing debate and improve patient outcomes in managing FIRCTs.

## Materials and methods

This retrospective comparative study was conducted at a single institution by a single surgeon. The study was approved by the hospital's institutional review board (IRB 202301070B0, Board Chairman Tsang-Tang Hsieh), and informed consent was obtained from all patients. The study follows the STROBE Statement. The indication of LTT transfer was FIRCTs defined by tendon retraction to the glenoid, fatty infiltration of supraspinatus and infraspinatus of grade > 2 according to Goutallier,^21^ and with a positive ER lag sign at 20° elevation in the scapular plane.^22^ The choice between Onlay and Inlay LTT was determined by the availability of Achilles allograft and autologous hamstring or peroneal longus autograft at the time of surgery. The exclusion criteria were irreparable subscapularis, true pseudoparalysis with shoulder forward elevation < 45°,^23^ dysfunctional deltoid, advanced glenohumeral osteoarthritis or cuff tear arthropathy (Hamada grade ≥ 4),^13^^,^^24^ and revision surgery. The tear condition of the subscapularis was evaluated using Lafosse classification.^25^ The study population comprised patients who underwent Onlay and Inlay LTT transfer from September 2019 to June 2022. All patients included were followed for at least two years postoperatively.

The primary aim of the present study was to assess and compare the outcomes of the two different LTT transfers, the Onlay and Inlay techniques. All patients had a preoperative and postoperative plain film of Anteroposterior (AP), axial view of X-Ray, and a thorough MRI. The authors evaluated preoperative and postoperative Range Of Motion (ROM), including Forward Elevation (FE), ER, Internal Rotation (IR), Acromiohumeral Distance (AHD), and Hamada classification.^26^ All radiographic data were interpreted by two independent observers: one radiologist and one orthopedic surgeon with >10 years of experience in musculoskeletal radiology and surgery. As for the radiographic data, the intra-rater reliability metrics were estimated using the Intraclass Correlation Coefficient (ICC) to evaluate the consistency between the raters. The authors recorded the American Shoulder and Elbow Surgeons (ASES) score,^27^ Single Assessment Numeric Evaluation (SANE) score,^28^ and the Constant score^27^ for the PROMs measurement. The PROMS were collected preoperatively and at least two-year follow-ups after the operation.

After evaluating overall outcomes, the authors conducted a subgroup analysis to compare the outcomes between the two techniques. The same radiographic parameters and PROMs were used to assess and compare the outcomes of the Onlay and Inlay LTT transfer techniques. The operative time and hospital stay were also compared between the two groups.

### Statistical analysis

Data were analyzed using IBM SPSS Statistics version 29.0 (IBM Corp., Armonk, NY, USA). Independent *t*-tests for normally distributed continuous variables and Mann-Whitney *U tests* for non-normally distributed continuous variables were utilized to compare the demographic and baseline characteristics between the Onlay and Inlay groups. Chi-square tests were used for categorical variables. For ROM, ASES, Constant Score, and SANE, Independent *t*-tests were conducted for normally distributed data and Mann-Whitney *U tests* for non-normally distributed data. A p-value of <0.05 was considered statistically significant for all tests. To determine the appropriate sample size for the study, a power analysis was conducted based on the mean and standard deviation of the primary outcome measures.

To determine the adequacy of the sample size for detecting a statistically significant effect, the authors conducted a power analysis for a two-sample *t*-test comparing the means of the experimental and control groups.^29^ The analysis was performed with a significance level (α) of 0.1 and a desired power of 0.6. The authors assumed an effect size (Cohen's *d*) of 0.7 based on prior research and the expected difference between the groups. Using the established parameters, the analysis indicated that a total sample size of approximately 30 participants is needed to detect the specified effect size with 60% power at the 0.1 significance level. Given the current sample sizes of 18 for the experimental group and 20 for the control group, the total sample size of 38 is adequately met.

To calculate the Minimal Clinically Important Difference (MCID) for the distribution-based approach. This approach involves calculating 0.5-times the standard deviation of the outcome measures to estimate the smallest change that is considered clinically significant. The authors first computed the change for each measure by subtracting the pre-intervention value from the post-intervention value. Specifically, the authors calculated the changes for ASES, SANE, and Constant scores. For each measure, the authors then calculated the mean of these changes to determine the MCID. The results are as follows: The MCID was evaluated by 0.5-times the standard deviation for the changes of the interested values. The MCID in ASES score is 12.23, the SANE score is 12.69, and the Constant score is 5.62. These values represent the average change observed in each measure, aiding in evaluating the clinical significance of the treatment outcomes.

### Surgical technique

#### Onlay LTT transfer

**Fig. 1:**
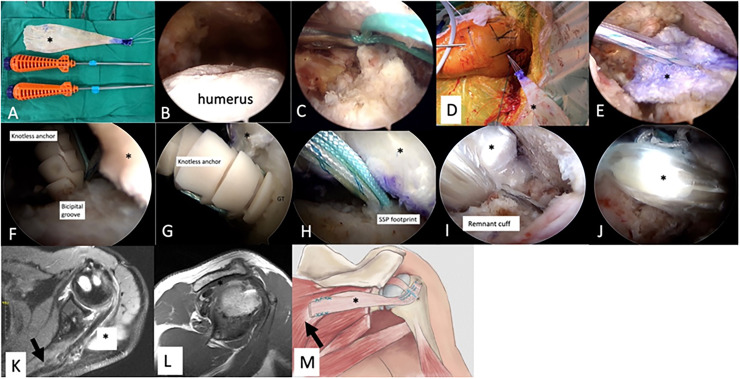
**Onlay LTT transfer technique of the left shoulder.** (A) The Achilles allograft was prepared with two locked sutures at the calcaneal part. (B) A diagnostic arthroscopy is performed to confirm the irreparability of the posterosuperior cuff tears. (C) Viewing from the lateral portal, one N°5 Ethibond suture is shuttled from the anterolateral portal through the interval between the scapular spine and infraspinatus fascia. (D) The suture exited the LTT harvest wound and tie with the two limbs of the locked sutures at the calcaneal part of Achilles allograft. (E) The Achilles allograft was shuttled into the shoulder joint through the interval between scapular spine and infraspinatus fascia. (F) One lateral row knotless anchor was inserted at the bicipital groove. (G) The other knotless anchor was inserted at the greater tuberosity. (H) Two to three suture-based anchors were used for multiple-point fixations at the supraspinatus footprint. (I) The posterior remnant cuff was repaired with the Achilles allograft. (J) After the fixation, the Achilles allograft covered fully the footprint of supraspinatus and infraspinatus. (K‒L) Follow-up MRI revealed the healing of Achilles allograft onto the footprint of the supraspinatus and infraspinatus, and LTT medially. (M) The whole construct is illustrated here. Asterisk, Achilles allograft; GT, Greater Tuberosity; Arrow, Lower trapezius.

The Achilles allograft was prepared with two locked sutures at the calcaneal part for lateral row fixation (Fig. 1A) at the back table. The patient is positioned in the beach chair position, and a diagnostic arthroscopy is performed to confirm the irreparability of the posterosuperior cuff tears (Fig. 1B). If the LHBT is present, an SCR with LHBT will be performed as Barth et al. proposed.^30^ LHBT is rerouted 5 mm posteriorly to the bicipital groove and fixed with a suture-based anchor by 2 lasso loops. After LHBT rerouting and fixation, LHBT tenotomy is done at the distal part of suture fixation, and the transverse humeral ligament is released for better visualization, facilitating lateral row knotless anchor implantation into the bicipital groove, which is believed to be the strongest part of the proximal humerus. Then the preparation shifts to the LTT harvest, as Elhassan et al. proposed.^18^

Viewing from the lateral portal, one N°5 Ethibond suture (Johnson and Johnson, USA) is shuttled from the anterolateral portal through the interval between the scapular spine and infraspinatus fascia (Fig. 1C) and exited to the LTT harvest wound. The two limbs of the locked sutures at the calcaneal part of the Achilles allograft were tied with the Ethibond suture and then inserted into the shoulder joint through strategic portals (Fig. 1D‒E). Two lateral row knotless anchors (4.5 mm Footprint Ultra, Smith & Nephew, Andovar, MA) were inserted at the bicipital groove (Fig. 1F) and greater tuberosity (Fig. 1G). Two to three suture-based anchors were used for multiple-point fixations at the supraspinatus footprint (Fig. 1H). The next phase of the procedure entails attaching the LTT to the Achilles allograft at maximal external rotation and 60 degrees of abduction.^31^ The posterior remnant cuff was repaired with the Achilles allograft in all patients in the Onlay group (Fig. 1I). Finally, the Achilles allograft covered the footprint of supraspinatus and infraspinatus fully and provided a spaced effect (Fig. 1J). The patient is placed in an abduction/external rotation brace for at least 6-weeks after surgery.^18^ The follow-up MRI revealed the healing of the Achilles allograft onto the footprint of the supraspinatus and infraspinatus, and LTT medially (Fig. 1K‒L). The whole construct is illustrated in Fig. 1M

#### Inlay LTT transfer^20^

**Fig. 2:**
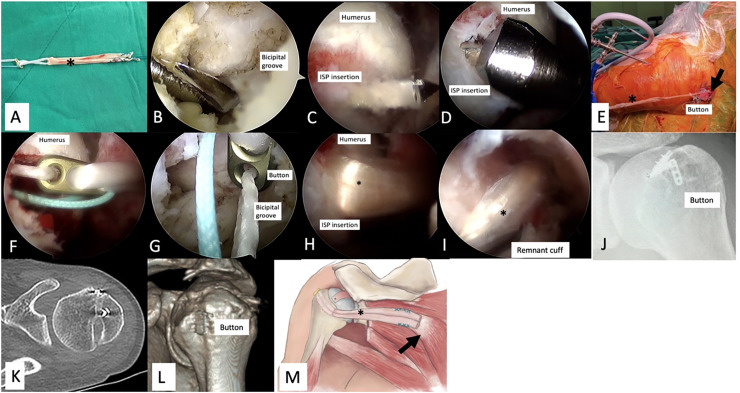
**Inlay LTT transfer technique of the left shoulder.** (A) The autograft is fixed with an Endobutton. (B) A temporal guide pin is first drilled from the anterior aspect of the humeral head at the bicipital groove. (C) The pin exits at the footprint of infraspinatus insertion. (D) An 8 mm drill was used posteriorly to anteriorly to create the desired humeral tunnel length for the tendon. (E) The free limb of the graft, not fixed with LTT, is passed through the loop of a 20 ‒30 mm EndoButton, and then shuttled intra-articularly from posterior to anterior. (F‒G) The Endobutton exits and fix at the bicipital groove. (H) The autograft is now entering posteriorly from the infraspinatus insertion site. (I) The posterior remnant cuff was repaired with the autograft in all patients in the Inlay group. (J, K, L) The postoperative X-Ray and computer reveals the Endobutton sits inside the bicipital groove. (M) The whole construct. Asterisk, Autograft; Arrow, Lower Trapezius.

In the Inlay LTT transfer technique, patients are placed in the beach chair position, which is like the Onlay technique. Simultaneously, the ipsilateral lower leg is prepared for the semitendinosus autograft harvest for male patients. For female patients, the Peroneal Longus Tendon (PLT) was harvested as the autograft (Fig. 2A) due to the cross-sectional size concerns. The LTT is marked and detached from the scapula spine, avoiding the spinal accessory nerve. The semitendinosus autograft (in females, PLT) is harvested in full length and fixed with the tendon part of the harvested LTT using a Krackow technique. If LHBT is present, an LHBT SCR is performed like in the Onlay LTT group. Finally, the transverse humeral ligament is released for better visualization and for button fixation.

A temporal guide pin is first drilled from the anterior aspect of the humeral head at the bicipital groove (Fig. 2B) and exits at the footprint of infraspinatus insertion (Fig. 2C). Then, a 4.5 mm drill is used to enlarge the humeral tunnel. An 8 mm drill is used posteriorly to anteriorly to create the desired humeral tunnel length for the tendon (Fig. 2D) and Endobutton (Endobutton, Smith & Nephew, Andovar, MA) passage. A suture shuttle is then passed through the tunnel and retrieved, facilitating suture and graft passage.

The free limb of the semitendinosus tendon (PLT in females), not fixed with LTT, is passed through the loop of a 20 mm or 30 mm EndoButton, and then shuttled intra-articularly from posterior to anterior (Fig. 2E), until it exits the bicipital groove (Fig. 2F‒G). The autograft is now entering posteriorly from the infraspinatus insertion site and fixed with the EndoButton anteriorly (Fig. 2H). Finally, the free limb of the semitendinosus tendon (PLT in females), not yet fixed with LTT, can be pulled backward to the desired tension and fixed side by side with the LTT using a Krakow suture. The posterior remnant cuff was repaired with the autograft in all patients in the Inlay group (Fig. 2I). The postoperative rehab program was the same as the Onlay LTT transfer group. On the postoperative X-Ray and computer tomography, the Endobutton sits inside the bicipital groove (Fig. 2J, K, L). The whole construct is illustrated in Fig. 2M

## Result

### Demographics and clinical characteristics

During the study period, a total of 47 patients received LTT, while 39 patients who underwent LTT transfer using either Onlay (*n* = 21) or Inlay (*n* = 18) technique were included. The mean age was 64 ± 5.9 years (64.5 ± 6.3 in the Onlay group, and 63.1 ± 5.3 in the Inlay group, *p* = 0.442). The cohort comprised 24 males and 15 females (*p* = 0.525).

In the Onlay group, there were six Hamada type 1, twelve Hamada type 2, and three Hamada type 3 patients, compared to eight Hamada type 1, ten Hamada type 2, and no Hamada type 3 patients in the Inlay group (*p* = 0.111). Regarding the subscapularis, eleven patients were normal, while eight, one, and one patient had Lafosse type 2, 3, and 4 tears in the Onlay group. There were eleven normal, one, five, and one patient who had normal, Lafosse type 1, 2, and 4 in the Inlay group (*p* = 0.518). All subscapularis could be restored and repaired to its anatomical position during the surgery. Regarding the LHBT, eight patients had a normal biceps tendon used as an LHBT SCR, four had a partial tear rendered tenotomy, and eight had a complete tear in the Onlay group. There were fifteen LHBT SCR, two tenotomy, and one complete tear of the LHBT in the Inlay group (*p* = 0.07). The average follow-up time was 25 ± 1 months in the Onlay group and 24.5 ± 0.9 months in the Inlay group (*p* = 0.07). The intra-rater consistency for related radiographic evaluations was calculated using ICC. For the Hamada score, the ICC was 0.88, and for the Lafosse classification, the ICC was 0.87, both indicating good reliability. All the demographic data are listed in Table 1.

**Table 1:** Patient demographics.

Variables	Onlay	Inlay	*P*-value	Total
Number	21	18		39
Age	64.5 ± 6.3	63.1 ± 5.3	0.442	64±5.9
Sex (M:F)	14:7	10:8	0.525	24:15
BMI	25.1 ± 2.9	26.1 ± 3.9	0.823	
Hamada score			0.111	
−1	6	8		14
−2	12	10		22
−3	3	0		2
Subscapularis			0.518	
Normal	11	11		22
Lafosse 1	0	1		1
Lafosse 2	8	5		13
Lafosse 3	1	0		1
Lafosse 4	1	1		2
Biceps			0.07	
BSCR	8	15		23
tenotomy	4	2		6
absent	9	1		10

BMI, Body Mass Index; LHBT, Long Head of the Biceps; BSCR, Biceps Superior Capsular Reconstruction.

Significant improvements in active FE and ER following surgery were observed in both groups. In the Onlay group, the active FE and ER improved from 118.6 ± 57.6° preoperatively to 145.7 ± 18° postoperatively (*p* = 0.046), and 31 ± 21.2° preoperatively to 44.3 ± 16° postoperatively (*p* = 0.027). In the Inlay group, the active FE and ER improved from 112.2 ± 49.2° preoperatively to 140.6 ± 30.6° postoperatively (*p* = 0.046), and 29.4 ± 27.1° preoperatively to 45 ± 16.9° postoperatively (*p* = 0.046). No significant differences were observed between groups regarding preoperative, postoperative, and gain in active FE and ER. However, the active IR did not improve in both groups following surgery. The AHD also did not change significantly after surgery. The preoperative and postoperative AHD were 6.3 ± 2.4, and 7.3 ± 2.5 mm (*p* = 0.229) in the Onlay group, while they were 5.2 ± 2.4, and 4.8 ± 3.2 mm in the Inlay group (*p* = 0.626). Interestingly, the postoperative AHD was significantly better in the Onlay group (*p* = 0.01). There was no progression of the cuff tear arthropathy according to the Hamada grade, and complications like retears of the graft, which rendered the revision surgeries between the two groups at the final follow-up. The interobserver correlations between the two observers for the assessment of the AHD and the Hamada grade were 0.8 (R2; *p* < 0.0001) and 0.88 (R2; *p* < 0.0001), respectively. There was no displacement of the Endobutton during the follow-up period in the Inlay group.

Regarding PROMs, there were substantial and statistically significant improvements in all scores following both LTT transfer procedures. In the Onlay group, the ASES score improved from a mean of 28.4 ± 5.1 preoperatively to 75.2 ± 7.4 postoperatively (*p* < 0.001). The SANE score also showed substantial improvement, with a preoperative mean of 25.2 ± 11.7, increasing to 76.9 ± 11 postoperatively (*p* < 0.001). The Constant score improved from 32 ± 9.7 to 76.3 ± 8.9 postoperatively (*p* < 0.001). In the Inlay group, the ASES score improved from a mean of 34.7 ± 8.8 preoperatively to 75.6 ± 7.7 postoperatively (*p* < 0.001). The SANE score also showed improvement, with a preoperative mean of 26.7 ± 13.3, increasing to 78.6 ± 9.7 postoperatively (*p* < 0.001). The Constant score improved from 36.8 ± 13 to 77.6 ± 8.6 postoperatively (*p* < 0.001). No significant differences were observed between the two groups regarding the preoperative and postoperative PROMs and the gain of PROMs, except the preoperative ASES was lower in the Onlay group (*p* = 0.009). Both groups reached MCID in all PROMs at the final follow-up. The preoperative and postoperative clinical and radiological results between the Onlay and Inlay groups were delineated in Table 2. The operative time (73.1 ± 5.7 vs. 74.1 ± 6.9 min, *p* = 0.634) and hospital stays (2.5 ± 0.6 vs. 2.3 ± 0.5 days, *p* = 0.289) were not significantly different between groups (Table 2).

**Table 2:** Preoperative and postoperative clinical and radiological and results between Onlay and Inlay groups.

	**Onlay**	**Inlay**	**p-value**
**Active FE, deg**			
Preoperative	118.6 ± 57.6°	112.2 ± 49.2°	0.716
Postoperative	145.7 ± 18°	140.6 ± 30.6°	0.518
p*-*value	**0.046**	**0.046**	
Δ FE, deg	27.1 ± 51.7°	28.3 ± 45.4°	0.94
**Active ER, deg**			
Preoperative	31±21.2°	29.4 ± 27.1°	0.847
Postoperative	44.3 ± 16°	45±16.9°	0.893
p*-*value	**0.027**	**0.046**	
Δ ER	13.3 ± 21.3°	15.6 ± 21.2°	0.747
**Active IR**			
Preoperative	12.5 ± 4.1	12.6 ± 3.7	0.98
Postoperative	13.4 ± 1.5	12.4 ± 2.4	0.134
p*-*value	0.346	0.917	
Δ IR	0.9 ± 4.5	0.1 ± 3.3	0.437
**AHD, mm**			
Preoperative	6.3 ± 2.4	5.2 ± 2.4	0.17
Postoperative	7.3 ± 2.5	4.8 ± 3.2	**0.01**
p*-*value	0.229	0.626	
Δ AHD	0.9 ± 3.4	0.5 ± 2.5	0.159
**ASES**			
Preoperative	28.4 ± 5.1	34.7 ± 8.8	**0.009**
Postoperative	75.2 ± 7.4	75.6 ± 7.7	0.894
p*-*value	**<0.001**	**<0.001**	
Δ ASES	46.8 ± 8.4	40.8 ± 10.9	0.061
**SANE**			
Preoperative	25.2 ± 11.7	26.7 ± 13.3	0.723
Postoperative	76.9 ± 11	78.6 ± 9.7	0.613
p*-*value	**<0.001**	**<0.001**	
Δ SANE	51.7 ± 15.3	51.9 ± 15.5	0.955
**Constant**			
Preoperative	32±9.7	36.8 ± 13	0.203
Postoperative	76.3 ± 8.9	77.6 ± 8.6	0.652
p*-*value	**<0.001**	**<0.001**	
Δ Constant	44.3 ± 11	40.8 ± 15	0.414

FE, Forward Elevation; ER, External Rotation; IR, Internal Rotation; AHD, Acromiohumeral Distance; ASES, American Shoulder and Elbow Surgeons score; ANE, Single Assessment Numeric Evaluation.

## Discussion

The present hypothesis was partially confirmed, that both the Onlay and Inlay techniques significantly improved postoperative clinical function, including active FE and ER, but not IR, as well as radiological assessment of AHD in patients with FIRCTs. Patients in the Onlay group did not show superior PROMs, and there were no significant differences between the two groups in terms of improvements in PROMs, including the ASES score, Constant score, and SANE score at the final follow-up. This suggests a positive effect on patient function and quality of life postoperatively for both techniques.

The overall finding of significant clinical improvement following LTT transfer, irrespective of the specific technique used, is consistent with the growing body of literature supporting this procedure for FIRCTs. Baek et al. divided patients into groups undergoing arthroscopic-assisted Latissimus Dorsi Transfer (aLDT) or arthroscopic-assisted LTT (aLTT) transfer, showing improved clinical outcomes for both treatments. However, the aLTT group outperformed the aLDT group in measures of shoulder ER, ASES score, activities of daily living requiring active ER, and AHD at the 2-year follow-up.^32^ Moreover, the progression of arthritis was lower in the aLTT group, suggesting aLTT may be a more effective treatment option for active, younger patients with posterosuperior irreparable rotator cuff tears. Baek et al. not only confirmed the benefits of LTT but also suggested its superiority over latissimus dorsi transfer in improving external rotation and maintaining AHD, reinforcing LTT’s role as an effective treatment.^32^

Similarly, the foundational work by Elhassan et al. using an Achilles allograft ‒ a technique comparable to the Onlay group ‒ demonstrated substantial improvements in pain and functional scores in a large cohort, even in patients with preoperative pseudoparalysis.^16^ The significant improvement in visual analog pain score, subjective shoulder value, and DASH score was noted in 90% of patients. However, patients with preexisting rotator cuff arthropathy (29 patients [71%] with Hamada grade 1 rotator cuff tear arthopathy, two patients [5%] with Hamada grade 2, and another two patients [5%] with Hamada grade 3) experienced persistent pain and restricted shoulder mobility, and two patients had a traumatic rupture of the transfer due to a fall. Despite some complications, aLTT transfer was found to be an effective treatment option for massive irreparable posterior-superior rotator cuff tears.

The present results mirror those of Almeida et al.,^33^ who also reported significant improvements in the ASES Score and pain after LTT transfer, despite using autologous hamstring tendons as their graft source. In their case series of ten patients, they observed significant improvements in the Shoulder Subjective Value, ASES Score, pain scores, and ROM at 6- and 12-months postoperatively. Similarly, the authors found that both the Onlay and Inlay techniques of LTT resulted in improved function in the patient cohort. However, it is worth noting that Almeida et al. used autologous hamstring tendon grafts, while the authors employed Achilles tendon allografts in the Onlay technique and semitendinosus autografts or peroneal longus tendon autografts in the Inlay group. Despite these differences in graft choice, both studies demonstrate the potential benefits of LTT in the management of irreparable rotator cuff tears.^33^

When comparing the Onlay and Inlay techniques, the present results suggested that both groups provide beneficial effects following surgery regarding active FE, ER, and all PROMs. Despite these comparable functional outcomes, a notable distinction emerged in the radiographic results, where the postoperative AHD was significantly better maintained in the Onlay group. The authors theorize that this superior AHD preservation in the Onlay group is multifactorial, stemming from both the graft properties and a modification to the original surgical technique. Biomechanically, the Onlay technique, which involves placing the Achilles allograft onto the footprint of the cuff, is thought to provide both a depressive force on the humeral head and a compressive force towards the glenoid. In contrast, the Inlay technique mainly provides a concentric force. However, both techniques provide a balanced horizontal force couple because all subscapularis tendons were intact or repairable, and the posterior force couple was reconstructed either with an Inlay or Onlay technique. Further studies are necessary to confirm this theory. The present findings align with the growing body of literature supporting the effectiveness of LTT transfer in managing patients with irreparable rotator cuff tears.^13^^,^^16^ These comparative results of the Onlay and Inlay techniques add a new dimension to this body of knowledge, providing valuable insights for surgeons when choosing the surgical technique.

According to the original technique by Elhassan et al.,^22^ SCR using the LHBT was not necessary in the Onlay technique. However, the authors choose to perform LHBT SCR whenever possible for two reasons. Firstly, it clears the space of the bicipital groove for lateral row anchor placement, confirming that the graft covers the most anterior part of the supraspinatus footprint. Secondly, it provides a greater spacer effect when the Achilles allograft is covered on top of the LHBT SCR. In the Inlay group, the lack of spacer effect provided by the thickness of the tendinous part of Achilles allograft may explain the difference in AHD at the final follow-up between the two groups. Although there are still debates on the optimal graft thickness used in SCR. Baek and Kim utilized a hybrid graft comprising fascia lata and dermal allograft and achieved an average 10.9 mm (range, 6‒19) graft.^34^ Although 62.1% of their patients achieve MCID, only 19% achieved a patient-acceptable symptomatic state. According to their data, an increased graft thickness and smaller preoperative AHD were significantly associated with postoperative discontinuity on serial MRI.^34^ In Jeon’s group, they reported the clinical outcomes in their SCR graft tear cases and found that the postoperative 1-year AHD is the most important independent risk factor indicating poor clinical outcomes after a graft tear post-SCR.^35^ Further studies on the role of AHD and its impact on clinical outcomes are needed to validate these theories, particularly since most existing evidence focuses on SCR rather than LTT.

Beyond the spacer effect, another critical difference between the techniques lies in the graft fixation method. The tubularized autologous hamstring or peroneus longus tendon inserts at a right angle into the proximal humerus, resulting in a 'killer turn' around the bony edge. This sharp turn has been shown to increase tensile forces on the graft, leading to elongation, potential failure, or tunnel widening.^36^ This is an inherent limitation of harvesting autologous hamstring or peroneus longus tendons without a bony plug, which is essential for reliable bone-to-bone healing. Fortunately, the authors did not observe displacement of the Endobutton during the follow-up period in the Inlay group.

### Limitation

Despite these promising results, the present study has certain limitations. First, the sample size is relatively small and from a single institution, which may limit the generalizability of the results. Also, based on the power analysis, the achieved power was below the commonly recommended threshold of 80%, which increases the risk of a Type II error, meaning that the study may have been underpowered to detect a true effect. Also, acknowledged that the preoperative ASES score was significantly different between groups and that the authors did not perform a multivariable analysis to control for this or other potential confounders. This was due to the relatively small sample size, which limits the statistical power of such models. The use of a distribution-based method for calculating the MCID is a valid but less robust approach than an anchor-based method. The absence of confidence intervals, which limits the interpretation of the precision of the effect size estimates, is again, the limitation of this study. Second, the retrospective design of the study may introduce some bias. Third, the choice of surgical technique (Onlay vs. Inlay) was not randomly assigned but depended on the availability of Achilles allograft and autologous hamstring or peroneal longus autograft. The non-randomized allocation based on graft availability and patient financial considerations is a primary source of selection bias. Fourth, the absence of postoperative imaging, such as ultrasound or MRI, to evaluate graft healing and tendon status may have provided additional insight into the structural integrity of the repair. Lastly, the minimum follow-up period of two years may not be sufficient to capture long-term outcomes and potential differences between the two techniques. Therefore, larger prospective randomized controlled trials with longer follow-up periods, comprehensive outcome measures, and postoperative imaging are needed to confirm these findings and to compare the long-term outcomes of the Onlay and Inlay techniques.

## Conclusions

Both Onlay and Inlay techniques of LTT transfer effectively improved shoulder function in patients with irreparable posterosuperior rotator cuff tears. The Onlay technique resulted in better AHD at final follow-up.

## URL address of the dataset

The datasets generated and/or analyzed during the current study are not publicly available due to patient privacy concerns and ethical restrictions but are available from the corresponding author on reasonable request.

## Authors’ contributions

Cheng-Pang Yang: Conceptualization; Investigation; Writing-original draft.

Tung-Lung Wu: Methodology; Validation.

Louis Yi Lu: Software; Formal analysis.

Lei Hsia: Investigation.

Poyu Chen: Data Curation; Visualization.

Joe Chih-Hao Chiu: Conceptualization; Methodology; Resources; Writing-review & editing; Supervision; Project administration.

## Funding

The authors gratefully thank the Taiwan Minister of Science and Technology and the 10.13039/501100005795Linkou Chang Gung Memorial Hospital for the financial support of this study (Grant: MOST 111-2628-B-182A-016, NSTC112–2628-B-182A-002, MOST110–2314-B-182A-019, NSTC 114-2314-B-182A-094, CMRPG391572, CMRPG3E0011, CMRPG5K0092, CMRPG3M2032, CLRPG3D0045, CMRPG5K021, SMRPG3N0011, SMRPG3P0011), and the Laboratory Animal Center, Chang Gung Memorial Hospital, Linkou.

## Data availability statement

The datasets generated and/or analyzed during the current study are available from the corresponding author upon reasonable request.

## Declaration of competing interest

The authors declare no conflicts of interest.
